# Cellular response of human apical papilla cells to calcium hydroxide and tricalcium silicate-based cements

**DOI:** 10.1186/s12903-021-01467-6

**Published:** 2021-03-09

**Authors:** Mauricio Garrido, Diego Morales, María Paz Saldías, Christian Fernández, Veronica Villalobos, Oscar Cerda, Mónica Cáceres

**Affiliations:** 1 Department of Conservative Dentistry, Faculty of Dentistry, Universidad de Chile, Santiago, Chile; 2 Program of Molecular and Cell Biology, Institute of Biomedical Sciences, Faculty of Medicine, Universidad de Chile, Santiago, Chile; 3 Millenium Nucleus of Ion Channel-Associated Diseases (MiNICAD), Santiago, Chile; 4 Faculty of Medicine, Universidad de Chile, Santiago, Chile

**Keywords:** Bioceramics, Apical papilla, Calcium hydroxide

## Abstract

**Background:**

This study aimed to evaluate the biological response of human apical papilla cells to different calcium hydroxide formulations and three tricalcium silicate-based materials.

**Methods:**

Primary cells were obtained from explants of young immature premolars. 20,000 cells adhered for 24 h over discs of Biodentine™, ProRoot®MTA, BioRoot®RCS and calcium hydroxide mixed either with sodium chloride 0.9%w/v or polyethylene glycol and UltraCal® were used to evaluate cell adhesion by scanning electron microscopy and cell viability by MTT assay.

**Results:**

Cells adhered to ProRoot®MTA showed an increase of F-actin like protrusions, suggesting bioactivity. Cells adhered to UltraCal® show protrusion such as filopodia. On the contrary, cells adhered to BioRoot®RCS showed no signs of any cellular protrusion. Regarding viability between the materials, we found a higher percentage of viability in cells cultured over discs of Biodentine™ and ProRoot**®**MTA.

**Conclusion:**

ProRoot**®**MTA and Biodentine™ exhibit a better cellular response of human apical papilla cells in vitro conditions compared to BioRoot® and calcium hydroxide diluted in sodium chloride.

## Introduction

Treatment of immature necrotic permanent teeth due to trauma is a clinical challenge mainly because of the lack of root development and the damage to the periapical area comprising Apical Papilla and Hertwig’s epithelial root sheath (HERS) [1]. Thus, a fragile root structure and the lack of apical foramen closure are the most common challenges [2]. Among the traditional approaches, apexification and one-visit apexification are the most commonly performed treatment methods [3]; with one-visit apexification being the more recommended method for traumatized teeth [4]. In recent years, regenerative endodontic procedures (REPs) have been proposed as a method of treatment for this clinical condition [5]. In either of these treatments, a calcium hydroxide (Ca(OH)_2,_ or CH) medication is widely used to significantly reduce the bacterial load promoting the healing of the periapical tissues [4, 6].

One-visit apexification involves in the creation of an artificial apical barrier to seal the open apex using a biocompatible material such as a tricalcium silicate-based cement, also known as hydraulic cements, to seal the open apex [7]; prior a CH medication for at least one week. CH, a strong base with a high pH, when is in contact with an aqueous solution, dissociates into calcium and hydroxyl ions inducing a hard tissue formation and antimicrobial activity [8]. On the other hand, REP is an approach based on the presence of the apical papilla and HERS; this therapy disinfects the root canal and then promote a blood clot formation that provides growth factors and cells from the apical papilla, the last step of this therapy being the tight seal of the cervical area of the tooth with a silicate-based cement [9].

Tricalcium silicate cements is a group of hydraulic cements with tri and dicalcium silicates as their chief ingredients, which react with water to form calcium silicate hydrate and CH, and release calcium and silicon ions, among others [10, 11]. An advantage of these materials is their ability to form hydroxyapatite, creating a bond between dentin and the tricalcium silicate cement, which is useful in obtaining a tight seal during apexification and REPs. It also has been proven that hydraulic cements induce dental pulp stem cell differentiation through the regulation of signal molecules, pathways, receptors and transcription control systems [12].

Apical papilla, a connective tissue located apically to the epithelial diaphragm, has been proved to play a key role in root development even in teeth with necrotic pulps [13, 14]. It contains less blood vessels and mesenchymal stem cell (MSCs) than the apical cell-rich zone but proliferates 2 to 3 folds than other cells of pulp [15]. Among these cells, the presence of mesenchymal stem cells known as stem cells from the apical papilla (SCAPs) have been proved, which show a heterogeneous nature by co-expressing Bone sialoprotein, Osteocalsin, Matrix extracellular phosphoglycoprotein, among others and a low percentage of STRO-1- positive cells with a high percentage of cells staining positive with osteo-dentinogenic markers in cultures [16]. Furthermore, SCAPs show a positive stain for neural markers such as Glutamate decarboxylase, Neuronal nuclear protein, nestin and neurofilament M. Because of its localization, apical papilla remains in contact with CH medication every time an apexification or a REP procedure is performed; in addition, its cells will maintain continual contact with the apical and cervical barrier of the silicate-based cement.

The cellular response could be defined as events within the cell that ultimately change its behavior requiring to adapt, interact with and often modify their surrounding extracellular matrix (ECM). This interaction is mediated by adhesion complexes that act as anchorage sites and enable the coordinated regulation of plasma membrane dynamics, by providing a physical link to, and through the active remodeling of actin cytoskeleton. One of the actin membrane protrusions is filopodia, which are thin, finger-like and can extent out from the cell edge [17]. Filopodia have been described to play a key role tethering, environment sensing and navigation [18].

The aim of this study was to evaluate whether there is a biological response of apical papilla primary cells from human immature young teeth to different CH formulations and three tricalcium silicate-based materials, which are commonly used in apexification and REPs.

## Materials and methods

### Apical papilla cell culture

Premolars with incomplete rhizogenesis from patients with an indication for extraction due to orthodontic reasons were collected to assess the cellularity of the pulp chamber, root canal and apical papilla zone. To acquire primary apical papilla cells, explants were obtained from apical papilla adhered to the root of the tooth from lower second premolars at 8 Nolla’s stage of tooth calcification. (n = 6). All donors signed an informed assent, and an informed consent was signed by the guardian of the pacient, in agreement to the Ethical Committee, Faculty of Medicine, Universidad de Chile (protocol 011–2018). All procedures were performed in accordance with the protocol. Cells were cultured in Dulbecco’s modified eagle´s medium (DMEM) (Gibco BRL, Grand Island, NY) containing 10% fetal bovine serum (FBS) (HyClone Laboratories Inc., Logan, UT), and Normocin (Invivogen, San Diego, CA) at 37 ºC in a 5% CO_2_ atmosphere. All experiments were performed using cells expanded between passages three and eight.

### Histology of cellularity of immature teeth

Teeth (n = 5) were fixed in 4% w/v formaldehyde (freshly prepared from paraformaldehyde Sigma-Aldrich #158127), decalcified in ethylene-diamine-tetraacetic acid 10% w/v for 3 months, stained with hematoxylin and eosin, and scanned using a Nanozoomer -XR digital slide scanner C12000 (Hamamatsu Photonics KK, Hammamatu city, Japan). Teeth were axially divided into 5 portions: apex (including the apical papilla zone) and 2 mm, 4 mm, 7 mm and 13 mm from the apex. Cross sections of 5 μm were then obtained from each portion.

The expression of STRO-1 was analyzed by immunohistochemistry. Antigen retrieval was performed by incubating the tissue samples with buffer containing Tris–EDTA pH = 8 for 30 min at 80 °C. The slides were then treated with 3% v/v hydrogen peroxide solution for 20 min and blocked with 10% v/v calf serum, 0.3% v/v Triton X-100 diluted in PBS for 2 h. Using primary antibody against STRO-1 (R&D system, MAB 1038) overnight at 4 °C [19]

### Cell adhesion by scanning electron microscopy

Discs of Biodentine™ (Septodont, Saint-Maur-des-Fossés Cedex, France.), ProRoot®MTA (MTA-mineral trioxide aggregate) (Dentsply, Jhonson City, TN, USA), BioRoot® (Septodont, Saint-Maur-des-Fossés, France), Ultracal (Ultradent, South Jordan, UT, USA. were prepared according to the manufacturer’s instructions and CH (Hertz, Santiago, Chile) mixed either with NaCl 0.9% w/v or polyethylene glycol (PEG) and incubated during 4 h at 37 °C over a crystal of 12 mm, pH was measured using pH-Stick 0–14 (Isolab, Wertheim, Germany) after one hour of contact between the biomaterials and DMEM (pH 7,4). Then 20,000 human cells from the apical papilla were adhered using DMEM 10% FBS for 24 h at 37ºC in a 5% CO_2_ atmosphere. Consecutively cells were fixed with 2.5% v/v glutaraldehyde during 4 h, and washed with sodium cacodylate buffer (0.1 M pH = 7.2). The samples were then dehydrated in alcohol, followed by critical point drying, metallized with gold and observed under scanning electron microscope (Jeol JSM-IT3LV, Tokyo,Japan).

### MTT assay

To perform this assay 20,000 human apical papilla cells adhered as a 50 μL drop during 1 h, over discs of Biodentine™, ProRoot®MTA, BioRoot®, Ultracal® XS and CH mixed either with NaCl or PEG were used, and a 24 well plate, then 500 μL of DMEM 10%v/v FBS were added to the well and cell viability was evaluated at 24 h and 72 h of adhesion between the cells and the biomaterials by the reduction of the tetrazolium salt, MTT, to form a blue formazan product (Sigma, St. Louis, MO, USA). Formazan crystals were solubilized with dimethyl sulfoxide and read it at 570 nm (Elisa reader Biotek Instruments inc, ELx-800, VT, USA).

### Statistical analysis

All experiments were performed at least four times on separate occasions using cells from different patients (n = 11; n = 5 for histology and n = 6 for cellular experiments). Statistical analysis was performed using the Mann–Whitney and Kruskal Wallis non-parametric tests. The statistical software Prism 5.0 from GraphPad (La Jolla, CA, USA) was used. In all analyses, *p* < *0.05* was considered to indicate statistical significance [19].

## Results

### Histological analysis of cellularity of cross and longitudinal sections of the teeth

**Fig. 1:**
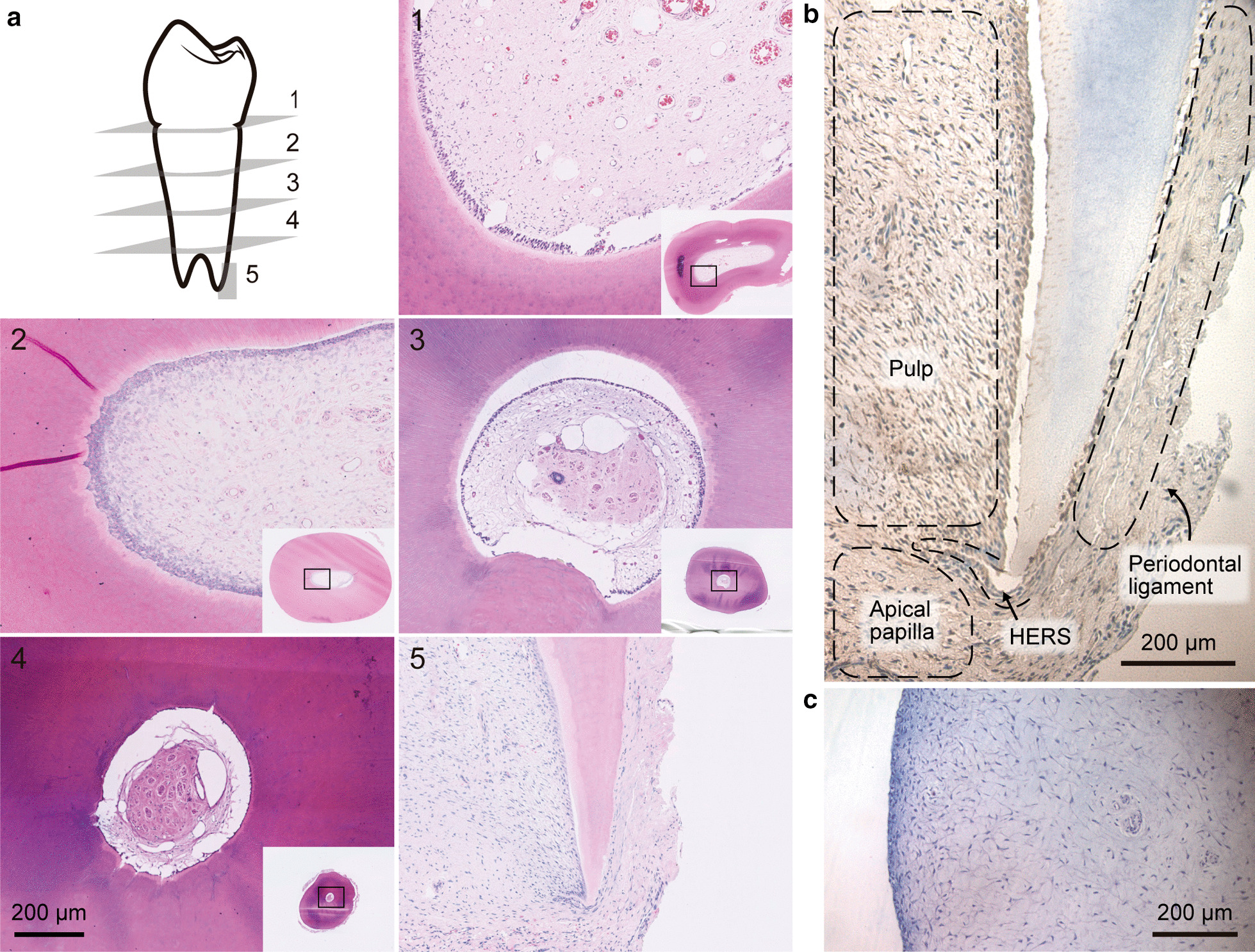
Histologic characteristics of immature premolar teeth. **a** Histological views of the longitudinal section of teeth (× 1.25 and × 7.5). **b** Chromogenic stain for STRO-1 in apical papilla. **c** Hematoxylin and eosin of extracted apical papilla (× 20) n = 5. scale bar 200um

Since one of the goals of treating an immature traumatized tooth is the early prevention of pulp necrosis by repairing the pulp tissue rather than performing invasive endodontic therapy, we studied the different cellular population in the young immature premolars. We analyze the specimens by hematoxylin and eosin staining five portions of the teeth (Fig. 1a). In the first section, which was a cross section of the cementoenamel junction (CEJ) where we could observe the largest volume of the dental pulp tissue, several blood vessels and different cell types like odontoblasts and fibroblasts (Fig. 1a). In the second to fourth portion, the volume of the pulp decreased compared to that in the previous section. In the fifth and longitudinal section, we observed the apical papilla, apical cell rich zone, periodontal ligament, HERS and pulp. Apexification and REPs are the more plausible treatment when dealing with a young immature necrotic tooth. Therefore, based on the fact that apical papilla cells come in contact with a calcium silicate-based cement in both the therapies, we evaluated the expression of a marker of mesenchymal stem cells like STRO-1 by immunohistochemical staining. As displayed in Fig. 1b there is a large number of positive cells for this marker in the apical papilla area, suggesting the presence of stem cells known as stem cells from apical papilla (SCAP). Hematoxilin and Eosin of extracted apical papilla (Fig. 1c).

### Cell viability of apical papilla cells cultured over CH and tricalcium silicate-based cement discs

**Fig. 2:**
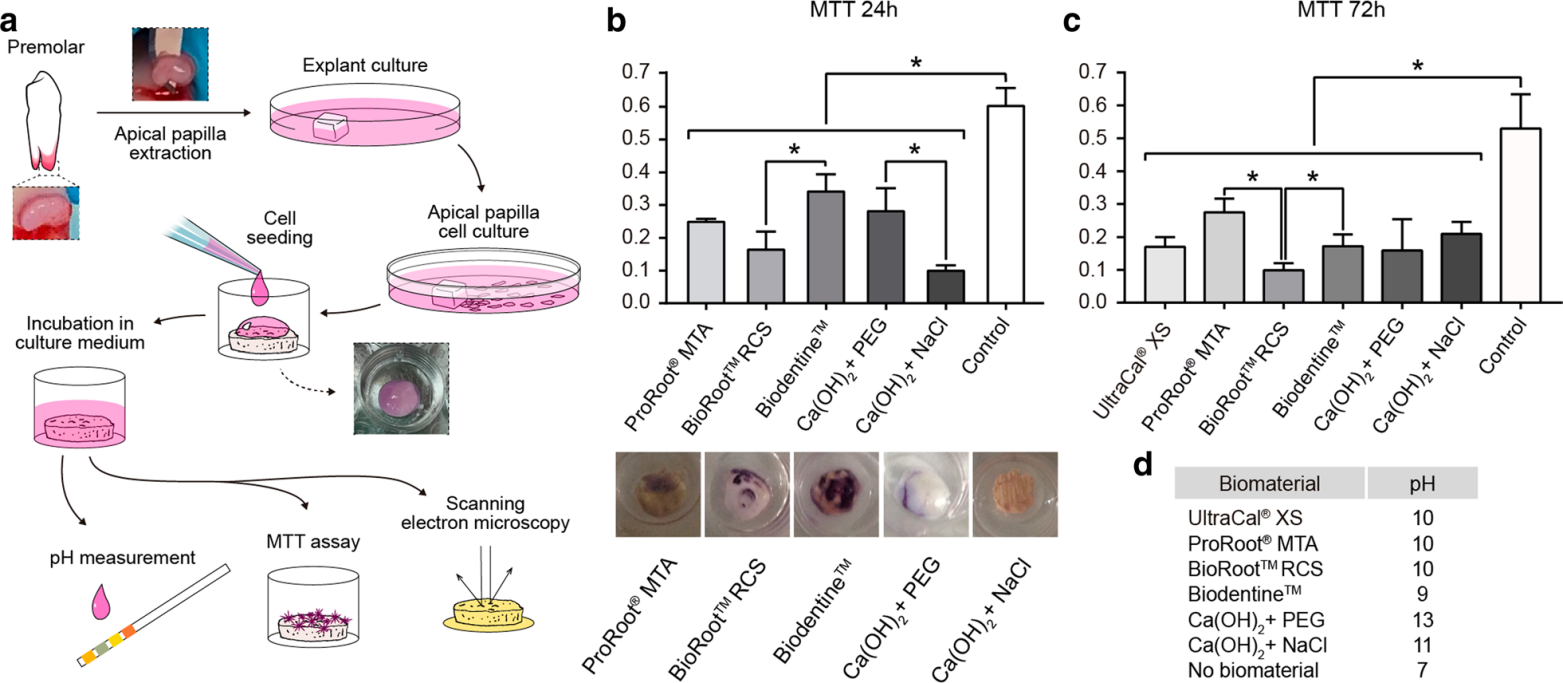
Viability of apical papilla cells to calcium hydroxide and tricalcium silicate-based cements. **a** Cartoon showing the methodology. **b** MTT assay: Graph indicates average and standard error of OD at 570 nm, Photograph of formazan crystal from cells adhered to the top of ProRoot®MTA, BioRoot® RCS, Biodentine™, CaOH_2_ diluted in PEG and CaOH_2_ diluted in NaCl 0.9% w/v. Apical papilla cells adhered to a 24 well plate was used as a control. The data are shown as mean ± SD n = 6 Asterisks indicate statistically significant differences. All materials are significant differences to control cells, and between the material significant different between Biodentine™ versus BioRoot® RCS p = 0.0317 and CH diluted in NaCl is significant diffrerent to: ProRoot®MTA (p = 0.0357), Biodentine (p = 0.0357) and CaOH diluted in PEG (p = 0.0095). **c** Quantification of MTT assay after 72 h of adhesion to, BioRoot® RCS, Biodentine™, Ultracal, CaOH_2_ diluted in PEG and CaOH_2_ diluted in NaCl 0.9% w/v. All biomaterial were significantly different to cells adhered to plastic, ProRoot®MTA versus BioRoot® RCS p = 0.0286, Biodentine™ versus BioRoot® RCS p = 0.0286 n = 5. **d** Table with quantification of pH

Cartoon showing the methodology from extraction of apical papilla and the final experiments (MTT and scanning microscopy) (Fig. 2a). The viability of apical papilla cells cultured on the top of the discs was evaluated through the reduction of the tetrazolium salt, MTT, to form a blue formazan product after 24 h and 72 h. The results for ProRoot®MTA, Biodentine™, BioRoot®RCS, both CH showed significant differences on cell viability after 24 h compared to control cells plated on a 24 well plate (Fig. 2b). Between the materials, Biodentine are significantly different compared to BioRoot®, CH diluted in PEG showed significant differences compared to CH diluted in NaCl (Fig. 2b). After 72 h of direct contact with the biomaterials, we observed that all biomaterials present significant differences compared to cells plated as a drop in a 24 well plate (control). Both, ProRoot®MTA, and Biodentine is significantly different compared to BioRoot®. Interestingly, after 72 h, CH diluted in NaCl 0.9% w/v and PEG have similar viability (Fig. 2c).

This data indicates that for cell viability of apical papilla cells, it is preferable to dilute CH in PEG than NaCl 0.9% w/v. Also, using PEG as solvent rather than water, can achieve an increase in hydroxyl ions release compared to water or saline. By adopting non-aqueous solvent such as PEG, greater dissolution and faster hydroxyl release can be achieved. [20]. Also, we quantify the pH of all biomaterials after one hour in contact with DMEM, showing the higher levels reached by CH diluted in PEG. ProRoot®MTA, Biodentine™, BioRoot®RCS present similar pH (Fig. 2d).

### Cell adhesion and protrusion enriched in actin to CH and tricalcium silicate-based cement as an indicator of bioactivity

Since a biomaterial is considered bioactive when it is not toxic for the cells and induces some cellular response, we evaluated the characteristics of cell adhesion and morphology, focusing on protrusion enriched in actin, like lamellipodia and filopodia. To evaluate cell morphology, we used scanning electron microscopy (SEM) to observe apical papilla cells that adhered to CH and tricalcium silicate-based cements for 24 h.

**Fig. 3:**
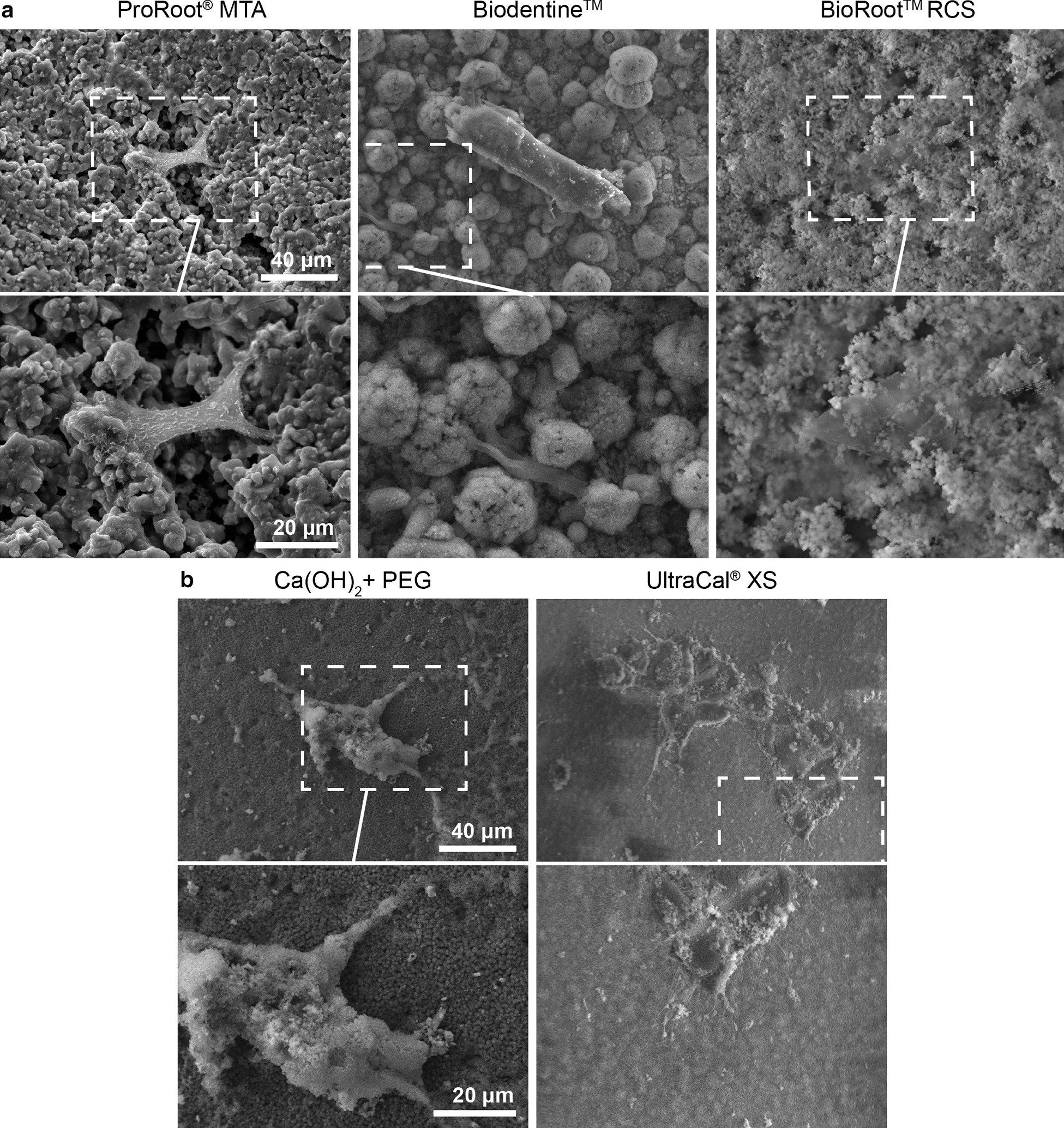
Apical papilla cells adhered to calcium hydroxide and tricalcium silicate-based cement. **a**. Scanning electron microscopic images of apical papilla cells adhered to bioceramics: ProRoot**®**MTA, Biodentine™ and BioRoot® RCS, **b** Apical papilla cells adhered to calcium hydroxide CaOH_2_ dissolved in PEG and UltraCal® XS. × 700 and × 1500. n = 5

Cells adhered to ProRoot®MTA show a morphology fibroblast-like, showing an adhesion like “a bridge” with a tight adhesion (Fig. 3a). We observed adhesion to Biodentine™ with no filopodia formation but we found a “*invadopodium*” protrusion from another cell. Also, cells cultured over BioRoot® did not present any F-actin protrusion.Using UltraCal® or CH diluted in PEG we observed an increase of filopodia formation, compared to the others CH pastes and to hydraulic cements. We did not find cells adhered to CH diluted in NaCl. (Fig. 3b).

**Fig. 4:**
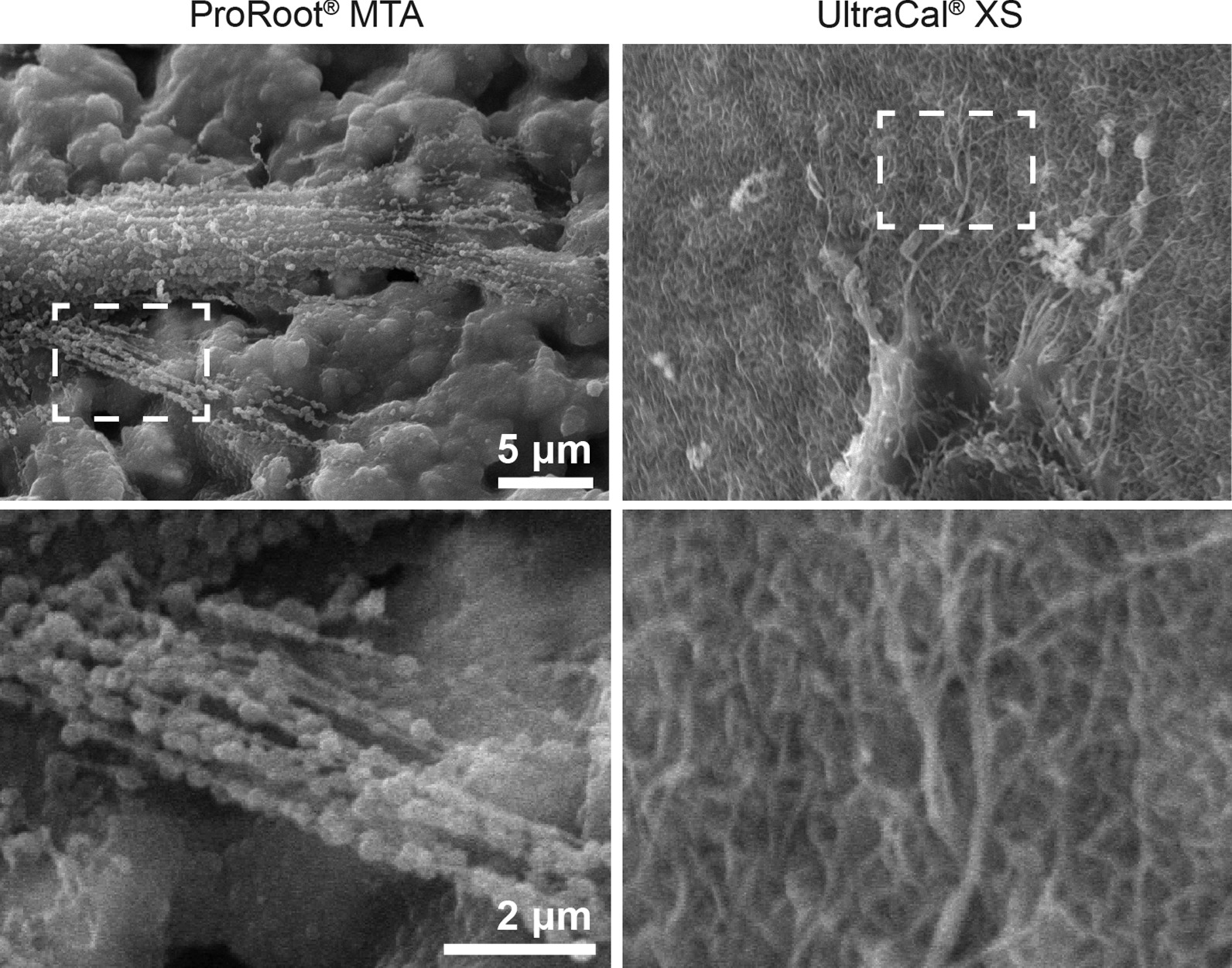
MTA and UltraCal® increased F-actin protrusion. A scanning electron microscopic images of apical papilla cells adhered to ProRoot®MTA, and UltraCal®. Scale bar 5 μm at × 1500. Scale bar 2 μm at × 4000. n = 5

In Fig. 4a, under higher magnification (4000x), we observed the filopodia formed at the end of the cells in the ProRoot®MTA disc, showing a globular structure like actin monomers. Using UltraCal® we observed many and long filopodia.

The formation of filopodia have been shown to be important for ECM sensing (stiffness and topology) and cell migration because cells migrating on planar substrates often display transient filopodia that are rapidly taken over by the advancing lamellipodium.

## Discussion

Evaluating the bioactivity of CH and tricalcium silicate-based cement it is of the utmost importance when dealing with necrotic immature young teeth treated with apexification or REPs since both therapies rely on disinfection obtained by CH and in the use of a tricalcium silicate-based cement as an apical plug or a cervical barrier respectively. Similarly, both therapies build upon the intimate connection of the abovementioned materials with cells from the apical papilla and in the impact that these biomaterials may have on viability and adhesion of these cells. In this study, we used human apical papilla cells cultured on discs of widely used materials in treatments of immature necrotic teeth, such as ProRoot**®**MTA, Biodentine™, UltraCal®, CH mixed with NaCl 0.9% w/v or PEG and the endodontic sealer BioRoot® to observe, analyze and compare the effects of these biomaterials on cell adhesion, morphology and viability. The use of primary cell cultures in direct contact with these biomaterials mimics a more accurate clinical scenario.

By immunohistochemistry, we observed the presence of STRO-1-positive cells in the apical papilla, which is indicative of a mesenchymal origin and is known to be a remnant of the dental papilla and it also has been proved to form a pulp-like tissue [21].

Analysis of the cell viability by the MTT assay demonstrated that ProRoot**®**MTA and Biodentine™ show the best performance compared with BioRoot®. Cell viability is important due the cervical or apical barrier of the hydraulic cement comes into contact with cells from the apical papilla: thus, it should stimulate the formation of a pulp-like tissue in the case of REPs and a cement-like tissue in apexification.

A recent study [22] using elute extracts of three hydraulic cements (ProRoot®MTA, Biodentine™ and PulpGuard) show that ProRoot®MTA and PulpGuard elicited cellular viability and, whereas Biodentine™ show a reduction on cell viability of human apical papilla cells after 48–72 h of treatment which is in contrast to our results. This may be because in our study, we used cells in direct contact with the bioceramics instead of elute extracts in order to imitate a more clinical scenario.

Regarding multi-visit apexification, it is well known that the apical barrier induced by CH is a more porous and prone to leakage since vascular inclusions can be found thus an inadvertent overfilling of the root canal sealer could reach the apical tissues thereby entering in contact with apical papilla cells [8]. Therefore, a bioceramic sealer as BioRoot® was included in our study. Another study concluded that BioRoot® is associated with better cytocompatibility compared with Endoseal MTA, which is in contrast of our results, maybe because the appointed study was conducted on human ligament cells [23].

Interestingly, evaluating cytotoxicity of root canal sealers is different when the material comes into direct or in indirect contact with cells. Most of root canal sealers show a cell viability decrease after four hours of direct contact, and when in indirect contact did not show any significant differences in cell viability [24]. Because of the above mentioned our research studied the response of cells cultured directly over discs of the material tested.

In relation to cell adhesion to the tricalcium silicate cement which is a good indicator of bioactivity and performance of that material, we probed for the first time the presence of protrusions enriched in actin like lamellipodia and filopodia in human apical papilla cells cultured over bioceramics. Moreover, tight adhesion between those cells and ProRoot®MTA were identified by SEM. The above is vital in REPs and apexification therapies since a biomaterial comes into contact with cells from the apical papilla and by doing so the response of these cells to different materials plays a key role since these materials should induce a tight seal and a biological response such as tissue formation.

Interestingly, based on the SEM study and MTT assay of this research using the whole population of apical papilla cells and on results shown in a clinical report in which a REP treatment was done using ProRoot® MTA [14], this material remains to be the gold standard for this therapy.

The clinical relevance of selecting the most biological interappointment medication and material for the cervical or apical plug is of the utmost importance when dealing with a necrotic immature tooth treatment since the prognosis depends on the bioactivity of these material once in contact with apical papilla cells and HERS.

## Conclusions

It was found that MTA and Biodentine™ induced a better cellular response from human apical papilla cells evaluated in vitro conditions by SEM and MTT compared with BioRoot®RCS and CH mixed either with NaCl 0.9% w/v or with PEG. When applicable, CH should be used mixed with PEG.

## Data Availability

The datasets used and/or analyzed during the current study are available from the corresponding author on reasonable request.

## Abbreviations

CEJ: Cementoenamel junction.
CH: Calcium hydroxide Ca(OH)_2_.
DMEM: Dulbecco’s modified eagle´s medium.
ECM: Extracellular matrix.
FBS: Fetal bovine serum.
HERS: Hertwig’s epithelial root sheath
MTA: Mineral trioxide aggregate.
MTT: 3-(4,5-Dimethylthiazol-2-yl)-2,5-diphenyltetrazolium Bromide or Tetrazolium salt.
PEG: Polyethylene glycol.
REP: Regenerative endodontic procedures.
SCAP: Stem cells from the apical papilla.
SEM: Scanning electron microscopy
